# Are we being drowned in hydration advice? Thirsty for more?

**DOI:** 10.1186/2046-7648-3-18

**Published:** 2014-10-29

**Authors:** James David Cotter, Simon N Thornton, Jason KW Lee, Paul B Laursen

**Affiliations:** 1 Exercise and Environmental Physiology, School of Physical Education, Sport and Exercise Sciences, Division of Sciences, University of Otago, PO Box 56, Dunedin 9054, New Zealand; 2 Faculté de Médecine, Université de Lorraine, U 1116 –INSERM-UL, 9, Avenue de la forêt de Haye, CS50-184 - 54505 VANDŒUVRE, Les Nancy cedex, France; 3 Defence Medical and Environmental Research Institute, DSO National Laboratories, Singapore; 4 Yong Loo Lin School of Medicine, National University of Singapore, Singapore; 5 Lee Kong Chian School of Medicine, Nanyang Technological University, Singapore; 6 High Performance Sport New Zealand, Auckland, New Zealand; 7 Sports Performance Research Institute New Zealand (SPRINZ), Auckland University of Technology, Auckland, New Zealand

**Keywords:** Dehydration, Thirst, Water, Exercise, Adaptation, Renal

## Abstract

Hydration pertains simplistically to body water volume. Functionally, however, hydration is one aspect of fluid regulation that is far more complex, as it involves the homeostatic regulation of total body fluid volume, composition and distribution. Deliberate or pathological alteration of these regulated factors can be disabling or fatal, whereas they are impacted by exercise and by all environmental stressors (e.g. heat, immersion, gravity) both acutely and chronically. For example, dehydration during exercising and environmental heat stress reduces water volume more than electrolyte content, causing hyperosmotic hypohydration. If exercise continues for many hours with access to food and water, composition returns to normal but extracellular volume increases well above baseline (if exercising upright and at low altitude). Repeating bouts of exercise or heat stress does likewise. Dehydration due to physical activity or environmental heat is a routine fluid-regulatory stress. How to gauge such dehydration and — more importantly—what to do about it, are contested heavily within sports medicine and nutrition. Drinking to limit changes in body mass is commonly advocated (to maintain ≤2% reduction), rather than relying on behavioural cues (mainly thirst) because the latter has been deemed too insensitive. This review, as part of the series on moving in extreme environments, critiques the validity, problems and merits of externally versus autonomously controlled fluid-regulatory behaviours, both acutely and chronically. Our contention is that externally advocated hydration policies (especially based on change in body mass with exercise in healthy individuals) have limited merit and are extrapolated and imposed too widely upon society, at the expense of autonomy. More research is warranted to examine whether ad libitum versus avid drinking is beneficial, detrimental or neither in: acute settings; adapting for obligatory dehydration (e.g. elite endurance competition in the heat), and; development of chronic diseases that are associated with an extreme *lack* of environmental stress.

## Background

The purpose of this paper is to critique the case for self-determined (largely ad libitum) versus institutionally advocated hydration behaviour acutely and chronically, with particular regard to humans moving in extreme environments. The major circumstance that might come to mind is dehydration through sweating during work or exercise in hot or humid environments, wherein daily turnover of water can exceed 12 L but varies tremendously [1,2]. Other environments may be problematic by virtue of their insidious nature and therefore also warrant consideration. These include the following: altitude-mediated dehydration by virtue of physiological and practical ramifications of high-altitude environments (hypoxia, low humidity and frozen); immersion-induced dehydration, particularly as might occur during open-water endurance swimming, notably during the increasingly popular 10 km and longer races held in sea water in tropical locations, and; perhaps also chronic low-grade, subconscious exposure to fluid dysregulation by way of a sedentary lifestyle in the man-made environment. That seemingly benign circumstance suffers from a notable lack of hydration research [3], but is complicated by related clinical conditions (e.g. diabetes, hypertension) and pharmaceuticals (diuretics and lithium-based anti-psychotic drugs). The main focus of this review is on exercise-related dehydration because it is widely relevant but controversial and topical. One intent with this review is to be provocative, to stimulate a critical re-examination of the literature on effects of dehydration and hypohydration and thus help direct further research in this field.

## Review

Dehydration refers to the process of losing water, which usually gives rise to the state of hypohydration (lower-than-normal body water). Many reviews are available on the acute and chronic physiological and performance effects of dehydration and hypohydration, e.g. [4-9], so information therein will not be repeated here except as it relates to the purpose mentioned above and the resulting questions expounded below. During physical activity, humans normally dehydrate to varying levels of hypohydration and fail to recover their mass deficit immediately following exercise despite ready access to fluids during and after exercise, a situation that has been referred to as both voluntary and involuntary dehydration [2,10,11]. We reiterate that 'hydration’ is not a simple notion of fluid balance; at a functional level, it concerns the volume, composition and distribution of body fluids, all of which are important and dependent on the timing, nature and extent of hydrative stress [3,9-14]. Indeed, the difficulty in measuring hydration is well recognised, and others have reviewed the complexity of its control and the errors inherent to its measurement [1,3,4,7,10,15-18].

### Key points

• Hydration refers simplistically to body water content, but functionally, it involves the volume, composition and distribution of body water, all of which are important but dynamic and difficult to measure collectively.

• This review critiques the case for ad libitum versus prescribed/imposed hydration behaviour in adverse environments, both acutely and chronically. Adverse includes those environments that insidiously lead to undesirable outcomes, acutely or chronically. General reviews of the physiology of fluid regulation in humans and the effects of hypohydration, hyperhydration and hyponatraemia are available elsewhere, e.g. [1,3,17,19].

### 1. What dangers are inherent with fluid-related stress?

*Acutely*, water is essential for physiological function at the molecular, cellular and systemic levels [1,4]. For example, it is: The medium in which metabolism occurs; a reactant and a product; the basis by which the volume of cells, tissues and organs is maintained; a shock absorber (e.g. for the brain); the medium for the mass-flow transport of gases, substrates, heat, hormones etc.; a thermal reservoir with a uniquely high specific heat capacity, thereby being capable of accepting or releasing large amounts of thermal energy with little change in tissue temperature, and; the substrate for evaporative cooling via sweating, which helps give humans an unparalleled versatility for moving in hot environments. Suboptimal physiological, mental and physical function and ultimately death can ensue through either excess or inadequate intake of water, but in the absence of medications and pathologies that cause dysregulation of fluid homeostasis, *inappropriate behaviour* or *insufficient availability* of potable water (and salt) is the essential feature underlying these extremes.

*Chronically*, low-grade mild hypohydration *possibly* contributes to suboptimal adaptation to repeated bouts of stress (i.e. training or heat acclimation) and to health impairment. For example, hypohydration appears to contribute to urolithiasis (development of kidney stones) [20], chronic kidney disease [21] and possibly also metabolic disease by way of metabolic effects of the principal fluid-conserving hormones, but the latter is speculation in the absence of appropriate human studies. These possible outcomes are discussed below.

**Figure 1:**
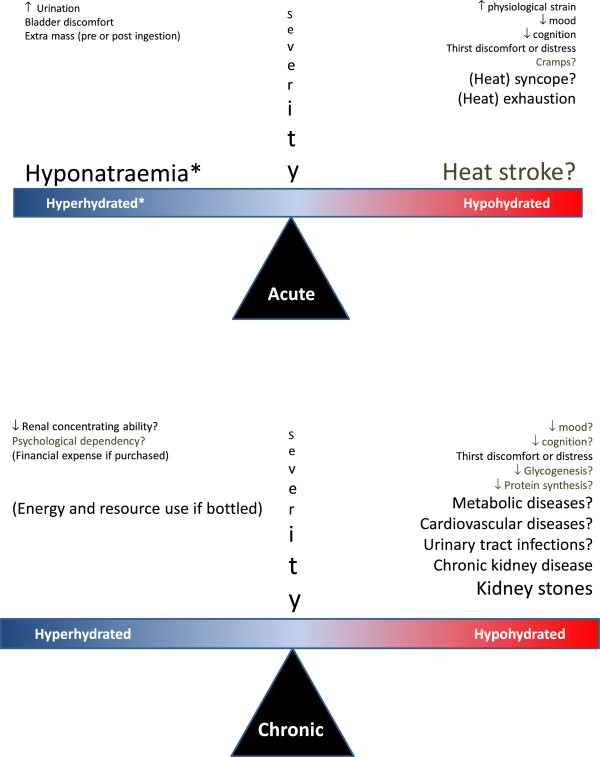
**Personal and societal effects of acute/chronic consumption of water above/below that required for fluid homeostasis.** The *three incrementing font sizes* denote outcomes causing a nuisance, morbidity and potential mortality. *Outcomes with question marks* are those for which we are not aware of any direct supporting evidence for humans behaving autonomously. *Asterisk* denotes that hyponatraemia can occur without hyperhydration per se, due to excess water relative to sodium content. The longer lists for hypohydration are not intended to convey higher relative importance. For example, hyponatraemia may be implicated in multiple adverse outcomes chronically (see [24-26]).

The salient issue is whether humans—individually or societally—are acutely or chronically at increased risk of harm from drinking ad libitum or from drinking avidly based on beliefs about appropriate hydration practice during exercise and other physical activity, or in relation to a healthy lifestyle. In view of the strong influence of the Internet and commercial interests [22,23], it is interesting to note that the search string 'The danger of dehydration’ retrieves approximately 160,000 hits in Google. Potential acute and chronic risks are shown in Figure 1 and discussed below.

### Acute hypohydration

Hypohydration can increase several forms of physiological strain at rest and especially during physical exertion, including cardiovascular [27-30], thermal [29,30], oxidative [31], metabolic [30,32] and possibly immune [33]. At least some of these effects are reduced or absent with outdoor-activity/realistic airflows (e.g. for thermal and cardiovascular strain) [34-37], depending on the extent of airflow and hypohydration. The attenuating effects of airflow are important but inadequately researched for other forms of strain (e.g. metabolic, oxidative, cerebrovascular and immune). Whether the increased physiological and psychophysical strain promotes injury or illness is less discernible. It is commonly advised that dehydration should be avoided because it impairs mood, cognition, psychomotor skill and aerobic performance, and predisposes to heat illness [e.g. [7]]: however, we *question how forcefully this advice should be applied in the majority of environments and activities that humans engage in*, for five main reasons. First, as mentioned immediately above and below, the effects of hypohydration appear to be physiologically, psychophysically and behaviourally exacerbated in well-controlled but thereby also reductionist studies, yet much of the advice used to support the benefits of limiting dehydration stems from such studies. Second, the body mass losses experienced in the vast majority of exercise training and competition were only modest before the American College of Sports Medicine published its influential Position Stands on hydration, in which they advocated the complete avoidance of *any* reduction in body mass in exercise and subsequently ≤2% reduction. That is, before the widespread emphasis of an all-encompassing guideline, most people appeared to self-regulate adequately in exercise training and competition anyway [38,39].

Third, scepticism exists [23,40] with regard to the long-held supposition that because dehydration increases body core temperature—and increased core temperature predisposes to heat illness—then dehydration will correspondingly increase the likelihood of heat injury [2,41-43]. Such reasoning precludes the immense role of behaviour in physiological control and, to our knowledge, is not supported for people in free-living circumstances anyway [40]. Psychophysical strain increases concurrently to increased physiological strain and will help drive behaviour [44,45]. Indeed, when volunteers are substantively hypohydrated in field research experiments, they became so thirsty and behaviourally averse to engaging in their work [2] that they would be less exposed to exertional heat-stress-mediated hyperthermia. Similarly, in lab studies that impose substantive hypohydration, participants cease exercise earlier and at *lower* core temperatures than when euhydrated [7,46], thereby limiting their exposure when the usual factors such as drinking or high airflow are unavailable. While this involves a suboptimal state of physical and social well-being, at least it provides self-protection against one of the triggering factors for heat stroke. The other major triggering factor of heat stroke is systemic inflammation [47] and central effects of systemic inflammation drive sickness behaviour, including lethargy. Thus, humans appear well protected against inadvertent heat stroke, as is evident from its rarity despite billions of people engaging in physical activity and sporting competitions in various environmental and immunological circumstances. We must emphasise here that we are not attempting to belittle the potential for inflammation-mediated heat stroke or the potentially contributing role of hypohydration; we are instead attempting to highlight the ability of normal physiology and behaviour to protect oneself against life-threatening illness in hugely variable, stressful circumstances. Clearly, recent or current febrile illness is contraindicated for heavy work or exercise, especially in the heat, and it would be similarly ill-advised to begin work or exercise when moderately hypohydrated, especially in circumstances with limited opportunity for rehydration or autonomy in controlling the exposure. Thus, notable exceptions are in people without access to fluids or perhaps in certain military circumstances where autonomous behaviour is more constrained, but these are special cases rather than the norm for physical activity.

Fourth, it is difficult to interpret the data apparently showing that dehydration facilitates heat illness despite numerous assertions of such (e.g. reviewed in [40,48]). Most assertions refer only to review papers, heat exhaustion or 'exhaustion from heat strain’, which is problematic because exhaustion is a self-limiting and transient outcome of exertion-related heat stress that helps prevent the frank and far more serious illness of heat stroke [49,50]. A frequently cited finding is that 17% of 5,246 cases of heat illness were associated with hypohydration in military training (especially locations in the southern USA in the summertime) [51]. But, unless this was a differential diagnosis (which we do not know), the prevalence of hypohydration may have been as high among individuals who did not succumb to heat illness. Irrespective, those statistics also appear to show that most heat illness is not associated with hypohydration. Athletes have consistently been found to tolerate substantial hypohydration (>6%) during competitive exercise with no ill effects [52,53], and the association between dehydration/hypohydration and hyperthermia may be largely spurious in high-airflow exercise settings due to the effect of exercise intensity on both factors [48,52].

Fifth and finally, by emphasising the importance of dehydration in heat illness so strongly, there is an inadvertent risk that people will erroneously believe that euhydration will protect against heat illness and thus, it also becomes more likely for them or their subordinates to over-drink. Indeed, an overzealous approach to prevent dehydration, especially in warm humid conditions, may lead to a rare, but life-threatening illness associated with the opposite fluid balance extreme—dilutional hyponatraemia [54]. In summary, little evidence is available to implicate dehydration as an important mediator of heat illnesses in exercise settings, and the protective role of behaviour in these settings is not conveyed sufficiently.

#### Other potential risks of hypohydration

*Exercise-associated muscle cramps* are not thought to be caused by body fluid deficits of water or sodium content [55,56]. *Syncope* may be more likely with hypohydration, but is secondary to the effects of exercise and heat *per se* and may have little functional significance in exercise contexts [57]. *Impaired cognition and skilled motor performance* are possible effects, which would be functionally significant in occupational and sporting competition contexts. It remains unclear the extent to which normal self-limiting levels of hypohydration impair cognition acutely [8,58]. Even when tested without concurrent heat stress or exercise (which may exert their own complex effects; [58,59]), cognition has been found to be both reduced (at 1–3% hypohydration: [60-62]) and improved (at 5%: [63]). Thirst has been shown to moderate the effects of hypohydration on cognition, with impairment evident only in individuals who were thirsty [64]. This makes it difficult to interpret data on cognition from any study in which participants felt thirsty when hypohydrated, including recent and otherwise robustly controlled studies on the effects of mild (approximately 1.5%) hypohydration [65,66], but where thirst was unfortunately not reported or considered as a separate factor. Thirst-related symptoms (headache) were evident in mildly hypohydrated females but not males in those studies, whereas cognitive functions were unaffected in the females but visual vigilance and scanning memory showed impairment in the males (at rest but not during exercise). Since exercise promotes arousal, exercise might attenuate or remove adverse effects of hypohydration on cognition or mood [67], but this remains unclear [65,67]. *Mood*, particularly perceived fatigue and tiredness, has consistently been shown to be impaired during mild (1-3%) hypohydration in resting individuals [65,66,68-70], but the concurrent stimulation of thirst in these fluid-deprived individuals would ordinarily act to prevent these outcomes [70]. Hypohydration has also been shown to impair skilled performance and cognition of sport-specific tasks [62,71], but interpreting these findings is again confounded by potentially important factors such as placebo effects and distraction by thirst (Table 1). It therefore remains unclear as to how much hypohydration *per se* (independent of heat) impacts on cognition, mood and skilled motor performance, particularly in movement situations and in the absence of thirst (which stimulates drinking, thereby reducing hypohydration).

**Table 1:** Factors distinguishing the dehydration that occurs in many outdoor settings from that in hypohydration research studies

	**Exercise settings (esp. outdoors)**	**Studies on hypohydration**	**Comments**
Hypohydrated at start	Seldom	Some	Larger effects; see text and Figure 3
Hypohydration extent	Mostly <2% BM	Usually ≥2% BM	Larger effects; see text and Figure 3
Airflow	Usually high (e.g. >2 m/s)	Usually slow (e.g. <2 m/s) and partial coverage	Exponential relation to heat transfer; decreased *T*_sk_ and *Q̇*_sk_ req
Thirst	Usually self controlled	Usually not self controlled, nor reported in results	May interact with other factors in Table
Familiarised to, or blinded against, the psychological effect of intervention	Not applicable	Very rare	Placebo and familiarisation effects (see Figure 2 and [85])
Exercise pacing	Often autonomous	Often imposed, for part or all of exercise	Interactions with other factors in table
Motivation to perform	Higher?	Limited?	Interactions with other factors in table

Note that each of these factors can impair the validity of research findings for exercise occurring in outdoor settings, yet the findings of such research is largely used to produce hydration policies that are applied to the outdoor settings.

*T*_sk_: skin temperature.

*Q̇*_sk_: skin blood flow.

### Acute hyperhydration and hyponatraemia

In healthy humans, hyperhydration is mostly well tolerated and transient at rest, incurring only discomfort, the need for more frequent urination and sleep disruption. In contrast, hyponatraemia arising from a dilution of the extra-cellular fluid (ECF) with or without an excess of body water volume (hyperhydration) is the most obvious and dangerous effect of drinking beyond thirst during exertional and/or environmental stress. The risk is elevated among those who have ample opportunity to ingest fluid in excess of requirements but difficulty offloading it (i.e. reduced free water clearance). Predisposing factors include beginning exercise with low plasma sodium concentrations [72], lower absolute but higher relative intensity of exercise, older age, pharmaceuticals such as non-steroidal anti-inflammatory drugs (NSAIDs) or selective serotonin reuptake inhibitors, and especially higher-than-required levels of arginine vasopressin (as occurs in the syndrome of inappropriate anti-diuretic hormone secretion; SIADH) [73]. Like heat stroke, clinically significant hyponatraemia appears to be rare during exercise but can be fatal, especially if misdiagnosed. In Westernised society, hyponatraemia is rare (<2%) also in the general population [24] but is prevalent among elderly individuals and especially those who are hospitalised, attributable in part to SIADH [25]. The aetiology, epidemiology and risk factors of hyponatraemia are addressed by others [17,22,24,25,73-76], including causes and consequences of chronic hyponatraemia [77].

### Key points

• Acute dangers exist with both inadequate and excessive intake of water (relative to salt), but both extremes have neurological mechanisms preventing their occurrence in the vast majority of exercise and environmental settings in which healthy people have access to clean water and are free to drink ad libitum.

• Our interpretation of the literature on dehydration is that despite widespread advice regarding the acute dangers of dehydration, the findings have limited relevance to free-living individuals with access to food and water.

• More research is needed in ecologically valid settings, including more attention on the roles of afferent and efferent components of behavioural regulation.

### 2. What regulations are established, and why/how are they set?

There exist few hydration-related regulations *per se*, yet advisory statements and guidelines are widespread.

Various militaries have hydration regimes, dependent on the environmental conditions, and levels of physical exertion and protective clothing. The guidelines adopted by the US military during the 1980s (ingesting up to 1.8 L/h) were revised downward for hourly and total daily fluid volume and refined to factor in both endogenous and exogenous heat stress, in the late 1990s following a high incidence of cases of hyponatraemia. Interesting and insightful accounts of the development and revision of these guidelines are available elsewhere [78,79]. Irrespective of whether adoption of guidelines by individuals actually improves work tolerance or reduces injury or illness, those guidelines are valuable in providing operational guidance on the total daily volumes of fluid that need to be made available in different work and climatic circumstances [2]. To attenuate the prevalence of exercise associated hyponatraemia, the International Marathon Medical Directors Association has recommended water stations to be spaced at least 1.6 km apart.

There are two dominant views regarding fluid replacement during exercise. One states that people should drink to prevent no more than 2% 'dehydration’ (~body mass loss) during exercise in temperate and warm environments, and rehydrate to eliminate any mass deficits soon after exercise [5-7,46,80-82]. The other suggests that it is adequate to drink ad libitum during and following exercise and cautions against adverse consequences of over-drinking [17,23,48,83,84].

**Figure 2:**
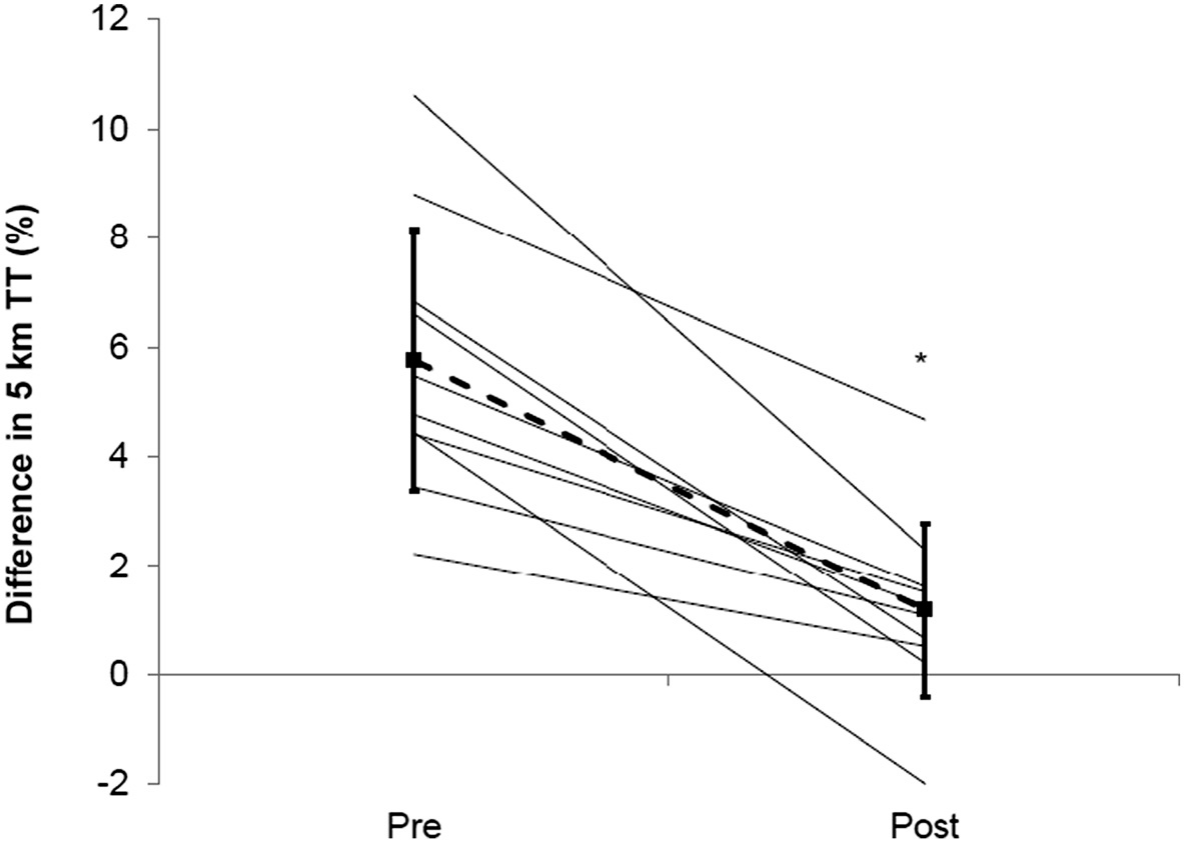
**Effect of hypohydration on exercise performance before and after familiarisation to the hypohydration.** Reprinted from Fleming J, James LJ. Repeated familiarisation with hypohydration attenuates the performance decrement caused by hypohydration during treadmill running. Appl Physiol Nutr Metab., 39: 124–129, Figure 3 (2013), with permission, © Canadian Science Publishing or its licensors.

**Figure 3:**
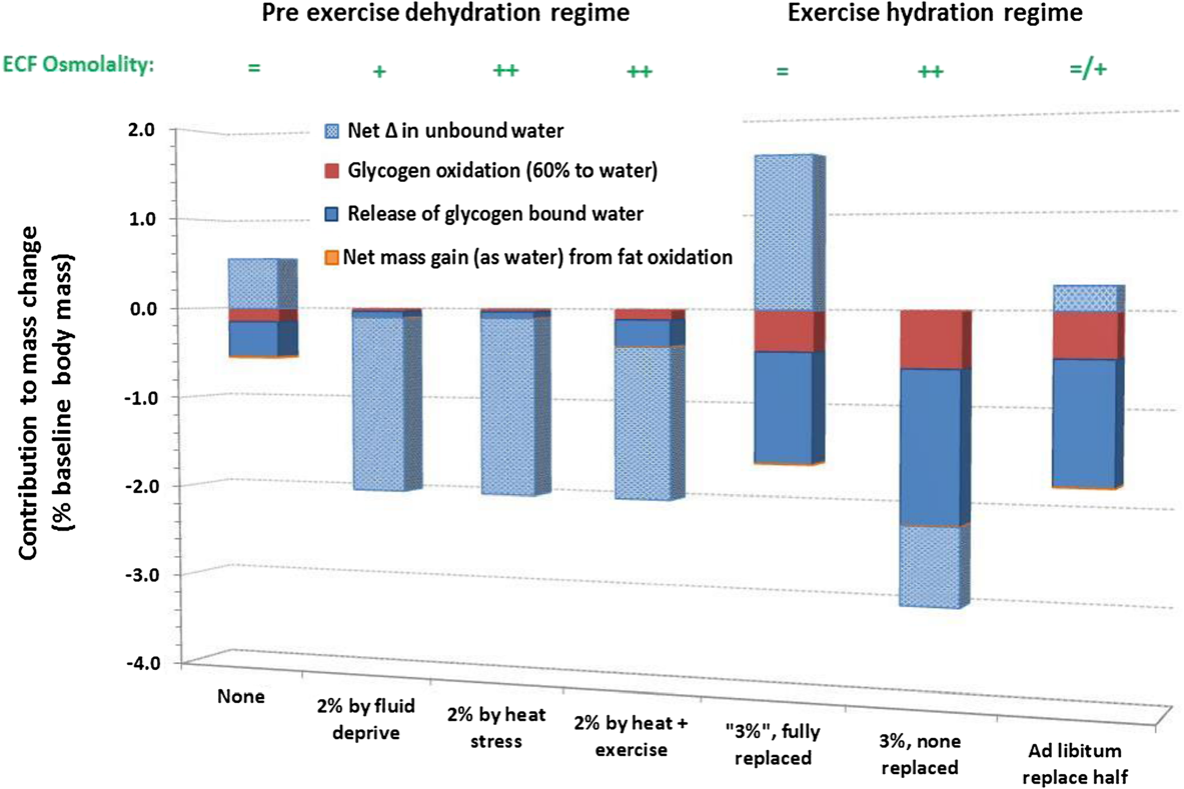
**Indicative contributions of different sources to changes in body mass for hypohydration induced before or during strenuous exercise.***Bar A* represents starting exercise euhydrated when rehydrated from an overnight fast (14 h), whereas *bars B–D* represent starting exercise 2% hypohydrated obtained as primary hypohydration (fluid deprivation alone over 24 h: *B*), heat stress alone (*C*) or light exercise in the heat (*D*). *Bars E–G* each represent strenuous intermittent or endurance exercise sufficient to oxidise 300 g of glycogen in a 70-kg person and produce 3% 'hypohydration’ (mass deficit), with full 'rehydration’ (3% mass restoration: *E*), no rehydration (*F*) or ad libitum rehydration (*G*; see [11]). Within the *bars*, 'Glycogen bound water’ (*solid blue*) refers to water that was previously complexed to and possibly within [94] glycogen before its oxidation. This contribution was assumed to be 2.7 times larger than the mass of glycogen oxidised, based on estimations in the literature of 3–4 times larger [95]. 'Unbound water’ (*stippled light blue*) refers to water that is not bound to glycogen molecules or created during oxidative metabolism. The mass difference from triglyceride metabolism is small (13% net gain, as water), so this component is difficult to see. A 10% energy deficit was assumed with 24 h of primary hypohydration [70]. An additional 111 g of glycogen oxidation in *F* versus *E* is based on measurements with 2–4% dehydration during exercise in temperate and hot laboratory environments [30,32], and an additional 30 g is estimated for *G* versus *E. Bars E* and *G* only show the appearance of not summating to 3% gross mass exchange because some of the ingested fluid would cancel out an attenuated mass of glycogenolysis-released water. See text for more interpretation of these differing circumstances and discussion of the implications, suffice to say here that the net volume of free water exchange depends on the hydration protocol used and thus needs to be considered when interpreting physiological, psychological and performance effects of dehydration studies.

*The prescribed view on hydration* has been promulgated most widely by the American College of Sports Medicine, whose position has been that mass loss should be avoided (pre 2007; [6]) or minimised to 2% body mass loss (since 2007; [7]), and rapidly eliminated following exercise. In many cases, this would mean drinking beyond thirst, both during and after exercise, as drinking ad libitum does not necessarily prevent such deficits during exercise or their rapid removal after exercise [10,11]. This prescriptive position on hydration is based on a substantial volume of literature showing increased physiological strain and reduced performance in studies wherein such losses were incurred before and/or during exercise. Yet, as shown in Table 1, several factors compromise the validity of those findings for most people exercising autonomously, especially outdoors. These factors affect the physiology and/or psychology of exercise performance, and yet we know of no study that has overcome all of these basic factors and still demonstrated an adverse effect of hypohydration on performance. Most of the studies used in substantiating the prescribed hydration policies have at least three validity problems affecting physiological or performance outcomes; unrealistically low airflow, no blinding to the hypohydration and no familiarisation to the stress of its imposition. The importance of familiarisation was recently demonstrated by Fleming and James [85], who gave participants four successive familiarisation exposures to 2% hypohydration and nullified the impairment in performance that it had otherwise caused (Figure 2), without diminishing cardiovascular strain. Similarly, low airflow impairs heat loss and raises skin temperature and vasodilation, thereby compounding cardiovascular strain, skin wettedness and discomfort. Effects of hypohydration on exercising heat strain and performance may occur partly by compounding the effects of warm skin [82,86], but whether its effects are necessarily adverse is not a simple matter. Increased plasma osmolality and hypovolaemia arising from sweat-induced hypohydration act centrally to reduce skin blood flow, which will aid in maintaining venous return, cardiac output and mean arterial pressure. The reduced skin blood flow would be more detrimental in conditions of low rather than high airflow over the skin, i.e. in those conditions used in most studies showing increased forms of physiological strain. In an often-cited study [28], Montain and Coyle (1992) had well-trained cyclists exercising for 2 h in the heat with moderate (2.5 m/s) airflow, and observed that cardiovascular and thermal drift were linearly related to extent of dehydration; even being larger with 2.3% than 1.1% dehydration (performance outcomes were not assessed). Unfortunately, however, their rehydration regime also provided carbohydrate replenishment, which may have suppressed neuro-endocrine stress responses and thus downstream indices of physiological strain. We found no such effect of dehydration on thermal, cardiovascular or metabolic drift for trained cyclists across 80-min exercise at higher exercise intensity and airflow (4.5 m/s) albeit in temperate conditions and approximately 2.5% final hypohydration, whereas drifts occurred in our untrained participants [35]. One study [87] has found that dehydration caused (slightly) more thermal strain in trained cyclists than untrained subjects, but this outcome may have arisen from the modest airflow (2.5 m/s) used in the face of their concomitantly higher work rates. Studies using realistic airflow in outdoor settings include trail running [88,89] and cycling hill climbing [90] and have shown increased thermal, cardiovascular and perceptual strain. However, these studies used prior hypohydration protocols, which would exacerbate the effects of hypohydration (see Figure 3). Performance effects are further confounded for additional reasons described in Table 1. Of the few studies that have attempted to address the psychological effects of having water deliberately withheld (i.e. as applies to almost all studies on dehydration), exercise-induced body mass loss of 2–3%, when incurred voluntarily by drinking ad libitum, has had no measurable effect on exercise performance [34,91-93]. When realistic airflow is then provided, the physiological effects of such deficits are also nullified or nearly nullified [34,37,91].

Another important factor in the validity of hydration research is how and when the dehydration occurs. Figure 3 shows the relative contributions of different sources to body mass changes for studies examining effects of dehydration on physiological, psychophysiological or performance outcomes. The four bars on the left side show sources contributing to the loss of body mass for studies using *pre-exercise* dehydration. Note that most of the mass loss during these interventions is free water (and also raises ECF osmolality) unless any exercise component is moderately stressful, and is hence physiologically expensive*.* Diuretic-induced dehydration, which is not shown in the figure, is wholly derived from this free water pool and particularly the ECF volume. Therefore, diuretic-induced dehydration (as used to 'make weight’ in weight-restricted sports such as rowing and wrestling or in anti-hypertension therapy) can incur even more strain and impairment during subsequent exercise [14,96].

The three bars on the right side of Figure 3 show the effects of three contrasting hydration regimes *during* exhaustive endurance exercise: (a) Full replenishment based on mass changes (as per [6]); (b) no fluid replenishment (as per many studies on dehydration) and (c) ad libitum drinking, which might typically prevent half the mass loss [37,38]. Note that these are theoretical proportions based on findings from a variety of studies [16,18,30,32,95]. An important caveat is that the contribution made by previously bound water is only theoretical. This fundamental contribution to mass loss in exercise has been acknowledged by others (e.g. [9,16,18]). Also unverified is the notion that the higher airflow with most exercise performed in the field will reduce glycogenolysis by virtue of less thermal and cardiovascular drift and sympathetic activation. Figure 3 nevertheless reveals several points relevant to interpreting the physiological, psychophysical and performance effects of hypohydration. First, hypohydration incurred prior to the exercise of interest would involve a larger proportion of free water loss than if it was incurred by virtue of competitive-intensity exercise, during that exercise. Second, the metabolic mass exchange profile is expected to be worse (more glycogenolysis and less FFA oxidation) during intense exercise with no fluid replenishment in laboratory than field conditions. Third, osmolality also increases more without fluid replacement, which independently increases heat strain and thirst, and would be rectified rapidly if opportunity was provided for ad libitum drinking [97]. Fourth, there seems to be no physiological rationale for attempting to achieve neutrality of body mass either during or following strenuous exercise until glycogen resynthesis is well established. For these reasons and those explained above, we believe that the literature on effects of hypohydration does not support prescribed hydration practices to the extent conveyed by its proponents. And, most importantly, ad libitum control of (functional) hydration status may be more accurate than is generally assumed.

*The ad libitum position on hydration during and after exertion*[17] is based on a different interpretation of the acute effects of self-determined dehydration and on safety against hyponatraemia relative to life-threatening effects of hyperosmotic hypohydration. Both of these states are prevented by *ad hoc* drinking behaviour in the majority of recreational and occupational settings. Both the pleasantness of drinking to satiate thirst and the displeasure of drinking when satiated have characteristic patterns of central nervous system (CNS) activation, with stronger activation during over drinking, especially in the motor cortex (suggested to reflect the extra effort required to continue drinking: [15]). Ad libitum drinking is not just a matter of drinking to thirst—and therefore, waiting until thirst begins—rather, it would indicate that hypohydration and hyponatraemia are constrained by some combination of factors driving drinking [77], such as habit (e.g. morning tea), thirst, comfort behaviour (e.g. carrying a bottle), anticipation and experience, social behaviour, availability of consumable and palatable liquids and the frequency of the need to void preferably in (clean) toilets (as described for kidney stone formers [98]). Athletes’ drinking behaviour appears to be additionally driven by a desire to avoid gastric discomfort [99].

The ad libitum position in an exercise setting is advocated also on the basis of observations such as the fastest runners generally finish the most hypohydrated [52,83,100]. But, this observation does not in itself validate the tenet that such performances would not be improved by more avid rehydration during exercise. Other factors do, however, further support this position: (i) the literature on ergolytic effects of dehydration may greatly over-represent its effect on actual performance, for several reasons, some of which are discussed elsewhere [23,34,84,35,93,101,102]) or illustrated in Table 1 and Figure 3; (ii) highly trained athletes may be less susceptible to the effects of hypohydration if tested in realistic airflow conditions [35,100], and this is not acknowledged adequately in hydration policies, and; (iii) absolute endurance performances in hot conditions (i.e. dehydrating and cardiovascularly-challenging; [103]) are so close to world records set in less dehydrating conditions (e.g. [53,104]) that the true effects of hypohydration must be smaller than is concluded from many lab-based studies. Finally, ad libitum drinking is at least as effective as drinking to prevent or limit mass loss to 2% [93,102,105,106], even in the heat [37,91], when airflow is realistic. Accordingly, the International Marathon Medical Directors Association (IMMDA) recommends that athletes drink ad libitum no more than 0.4–0.8 L/h.

### 3. Pros and cons of self versus prescribed acute exposure

Humans move in a myriad of benign and stressful environments for an immense variety of reasons, nearly all of which involve autonomous behaviour (including pace, pattern and duration of physical activity). Even in the specific cases of exercise *per se*, body mass loss seldom exceeds approximately 3% in team sports or 4% in distance running, but is mostly <2% whether in training or competition ([38,39]). Mass losses in exercise could not be considered hazardous and would mostly be self-limiting through behavioural responses to ingest water and salt or decrease output (see above). Therefore, we believe that ad libitum rather than prescribed drinking would suffice in most settings, for the reasons outlined above, with some caveats as noted below. Ad libitum may be even more appropriate when exposed to stressors that alter fluid regulatory control such that neutrality of body mass has additional validity problems—e.g. in ultra-endurance exercise or at altitude—as also noted below.

*Possible exceptions to ad libitum drinking:* Thirst is not stimulated appreciably until plasma osmolality rises by approximately 6–10 mOsmol/kg [70,97], although elevated angiotensin and reduced plasma volume provide additional stimuli [107]. Thus, pre-emptive and bolus drinking may be warranted to help limit obligatory hypohydration under conditions of constrained fluid availability or artificially high heat stress, e.g. ultra-endurance swimming in sea water, foot racing over large distances in arid land, or performing heavy work with encapsulation of the body or face. Pre-emptive hyperhydration is achieved more effectively with glycerol or sodium citrate and chloride solutes than with low-sodium fluid [108-111]. However, hyperhydration has shown only small benefits in attenuating physiological strain and improving work capacity during compensable heat stress and water deprivation [109,110,112,113], and no measureable benefit during uncompensable heat stress (for reviews, see [111,114]).

Newcomers to hot environments are susceptible to chronic hypohydration [2,41]. They may have a blunted drive to drink when hypohydrated because the higher sodium concentration in their sweat would blunt the rise in plasma osmolality and hence the stimulation of thirst [115]. Since rehydration occurs particularly at meal times in hot environments (appropriately) [2,41], permitting time to eat is important, and salt supplementation may be warranted for newcomers [81]. Heat-acclimated and aerobically trained individuals can dehydrate more rapidly by virtue of higher work capacities and sweating power, but they also have larger extracellular fluid volumes and develop stronger rehydration behaviour [107,116] and hence may not be at higher risk of problematic levels of hypohydration. Diarrhoea and vomiting also constitute special cases for aggressive replenishment of water and salt because of their potential to cause severe hypohydration without osmotic stimulation of thirst.

A more proactive approach to rehydrating from hypohydration appears warranted in the elderly due to an elevated thirst/osmolality threshold [117], less total body water (TBW; i.e. less volume reserve) and higher prevalence of risk factors for chronic diseases that may be exacerbated by hypohydration (discussed below). Finally, maintaining fluid balance during competitive ultra-endurance swimming especially in tropical locations is made difficult by factors that promote loss of sodium and water or constrain their intake. Sweat rates can exceed 1 L/h [118] alongside urinary losses that are higher than in terrestrial exercise due to the prone posture and hydrostatic pressure of water favouring higher renal blood flow and secretion of atrial natriuretic peptide, and less secretion of aldosterone. Swimmers also have limited opportunity to drink substantial quantities during competition. The hypohydration would presumably be more functionally important for swimming before terrestrial exercise (e.g. Ironman triathlon).

*Possible special cases for ad libitum drinking:* As exercise becomes prolonged, beyond approximately 8 h, plasma volume can expand isonatraemically to an extent that it eventually exceeds pre-exercise volume, in conjunction with increasing TBW volume, while fat mass can decline measurably [119]. The expansion seems to attain a consistent mean level of 20–25% across variable modes, patterns and intensities of upright exercise, initial haemoglobin concentration, aerobic fitness and environmental conditions [120-124]. The mechanisms involve water and sodium retention due to (orthostatic) stress-mediated secretion of aldosterone [120,125,126] and anti-diuretic hormone [123] causing expansion of the ECF volume, and albumin production selectively expanding the plasma volume [124]. The wider expansion of TBW with oedema has also been suggested to reflect an inflammation response [120]. In such cases of huge energy and water metabolism and shifts in fluid volume, ad libitum ingestion of food with water or sports drinks seems most appropriate, whereas reliance on sports drinks and/or maintaining body mass can be problematic [127,128].

High altitude and polar exploration also have complex effects on fluid balance, which are further affected by exercise and acute mountain sickness (reviewed in [129]). Practically, water availability can be constrained by its frozen state, while losses can be elevated even at rest due to low-humidity air, hypoxia-induced hyperpnoea and diuresis. Water and sodium losses are further increased during work due to disproportionate hyperpnoea and sweating. Plasma osmolality is elevated markedly at altitude without raising anti-diuretic hormone (ADH) or thirst [129]. On the other hand, SIADH occurs in perhaps one third of individuals upon acute exposure and appears causal in their higher acute mountain sickness scores [130]. Thus, although fluid balance may be more difficult to maintain at high altitude (and in polar environments), fluid regulatory control is altered and zealous drinking behaviour is not without risk.

### Key points

• Ad libitum drinking seems appropriate in most exercise and environmental settings, but in special circumstances of obligatory hypohydration, anticipatory drinking is warranted.

### 4. Can humans adapt? Is it meritorious? (Adaptations or maladaptations?)

*Can we adapt?* It is widely assumed that humans cannot adapt to the physiological or physical capability effects of hypohydration, on at least two lines of evidence. First, daily dehydration does not reduce fluid requirements during dehydrating exercise in the heat, irrespective of whether individuals are acclimatised to those conditions or not [2]. Second, acute hypohydration has been found to negate the thermal benefit of short-term aerobic training and heat acclimation [131] and interfere with hypothalamic and gene transcriptional adaptations to heat (in rats: [132]). Certainly, any adaptations are not as apparent as those from stressors such as heat and hypoxia. However, some adaptive potential might be anticipated on several bases [35,117]: (i) humans show adaptation to most other stressors; (ii) different components of fluid-regulatory control systems could adapt and have been found to do so (e.g. renal concentrating ability markedly increases with short-term (3-d) hypohydration and diminishes with over-drinking [133,134]); (iii) if hyperosmotic hypovolaemia increases other aspects of physiological strain (e.g. glycogenolysis), it may act as a synergistic conditioning stimulus, and; (iv) some individuals regularly experience such hypohydration by virtue of intense endurance exercise training, and cross-sectional data across fitness levels indicate that they have reduced sensitivity to its physiological and performance effects (as discussed above).

Some adaptation to repeated dehydration has been reported in response to 5–6 days of daily exercise in the heat causing 2–3% hypohydration [135,136]. In a controlled cross-over heat acclimation study, the acclimation-induced reduction in heart rate under standardised exercise heat stress tests was approximately 11 beats/min larger (*p* =0.05) following mild hypohydration compared with euhydration during acclimation bouts, and plasma volume expansion was approximately 4.5% larger (*p* =0.06) [135]. Core temperature was clamped during acclimation bouts to prevent any effect of hydration on the thermal stimulus. Other outcomes were unclear. In contrast to that study undertaken in aerobically trained men, forced water intake (double daily intake for 7 days) has been shown to improve acute heat tolerance of unacclimatised, untrained men and possibly enhance their acclimatisation to heat [137]. So, it is still unclear whether and to what extent adaptations occur in response to repeated hypohydration or attempted hyperhydration.

*Is adaptation meritorious?* There seems little merit in adapting to hypohydration for most individuals, unless repeated dehydration provides adaptations that are either ergogenic in their own right or aid fluid retention during some forthcoming exposure to substantive dehydration. The ergogenic issue is unresolved, so mild, self-regulated/limited dehydration during stress conditioning cannot be advocated at this time, but we believe that it cannot be discounted either [135]. Improving fluid regulatory control would be beneficial to athletes preparing for prolonged field, court or endurance competitions undertaken in hot and dehydrating conditions, in which pronounced dehydration is obligatory. However, the human studies that showed such renal adaptations used sustained and substantial hypohydration, which would be counterproductive for several reasons (e.g. cellular metabolism, anabolism, comfort and possibly hypothalamic effects; [138-140]).

Withholding availability of amino acids [141] but not water, electrolytes or carbohydrate [136] after bouts of training attenuates hypervolaemic responses to exercise [142], especially in older adults [143], and attenuates the higher rates of protein uptake into muscle following exercise. So, it seems likely that ingestion of at least the amino acids is important and perhaps water to reduce the catabolic hormone profile [144], although the catabolic/anabolic hormone profile in exercise recovery when hypohydrated is complex [145]. Furthermore, in vitro experiments indicate that muscle protein synthesis may be up or downregulated by hyperhydration or residual hyperosmotic hypohydration, respectively [138,140,146,147]. Another consideration is that a high protein intake requires more water to be consumed to eliminate the excess urea produced from the increased amino acid metabolism [148].

### Key points

• The fluid regulatory control and cardiovascular systems undergo strain due to the dehydration of exercise (with limited airflow) or environmental heat stress, some elements of which have shown adaptation with chronic exposure. The functional implications of mild and self-regulated dehydration—or, conversely, forced drinking—are unresolved.

• At least some rehydration concurrent with ingestion of amino acids following stress-conditioning bouts appears to be beneficial, especially for older individuals.

### 5. Pros and cons of self versus prescribed chronic/adaptive exposure

The seemingly basic issue as to whether humans are chronically in an optimal hydration status by virtue of ad libitum drinking behaviour is unresolved, as mentioned above (Figure 1) and discussed by others [149-151]. The data are sparse and conflicting. In healthy humans, over-drinking becomes unpleasant and requires additional cortical activation compared with drinking to rehydrate from hypohydration [15]. Therefore, death from hyponatraemia at rest is not evident from chronically drinking ad libitum or from the contrasting approach such as drinking according to the common doctrine of 8*8 (i.e. drink at least eight 8-ounce glasses of *water* per day) [149]. However, exacerbation of chronic hyponatraemia leading to a wide variety of other pathologies (e.g. osteoporosis [26]) and functional problems (e.g. poor balance [25]) may be of concern for elderly individuals, especially those who are hospitalised or on medications such as thiazide diuretics and selective serotonin reuptake inhibitors [25]. Another important consideration with large numbers of humans chronically drinking above ad libitum is that it demands more energy from finite resources for the manufacture and transport of water bottles because this approach to hydration is understandably promoted by the bottled water industry [152].

In contrast and speculatively in the absence of intervention studies in humans, chronic, low-grade hypohydration has been suggested as a contributory factor in type 2 diabetes and obesity and thus the metabolic syndrome, particularly for older individuals living sedentarily or frequently exposed to passive heat stress (e.g. living in urban heat islands in summer, with limited air conditioning). Cell culture studies have shown that increased hydration leading to increased cell volume increases cell signalling response to insulin [139,153]. Furthermore, in humans, increasing hydration using slightly hypoosmolar solutions increases whole body lipolysis [154]. In work using obese and normal rodents, treatments using inhibitors of the renin-angiotensin system increased water intake with an associated improved insulin sensitivity, increased energy expenditure and reduced fat mass [155-159]. These results *could* indicate increased hydration has a positive effect on cell metabolism, possibly through modulation of cell volume. Medications aimed at inhibiting the renin-angiotensin system are used extensively in the treatment of cardiovascular disease (>85% of treatments) as well as in the treatments of obesity, type 2 diabetes and cancer. Antagonists of the renin-angiotensin system are part of an effective treatment also in Alzheimer’s disease [160,161]. The presence in the blood of angiotensin indicates hypohydration, which may contribute to these relatively modern diseases for reasons explained above [13,162]. Any factor that exacerbates chronic hypohydration (and hence the angiotensin system) might promote these diseases, whereas factors that prevent these diseases, such as physical activity and eating more fruit and vegetables [163-165], also have a positive effect on TBW volume chronically*.* These lifestyle interventions are widely recommended, but their voluntary uptake is modest in the most affected countries [166,167].

### Key points

• Whether humans are generally hydrated optimally on a chronic basis is undetermined, but inhibition of angiotensin, an indicator of hypohydration, is beneficial in several diseases of sedentary living.

## Conclusion

### 6. Suggestions and future directions

A large literature exists on the physiological and work capacity effects of experimentally imposed and controlled hypohydration, at levels that have marked effects on physiology and performance. Such studies are valuable for reliably identifying mechanisms and dose/response relations [8,168]. The literature on the psychophysical effects of hypohydration (i.e. on mood, cognition and skilled motor performance) is even more adversely affected by the lack of blinding and added difficulty in identifying underlying mechanisms [15,58]. A small and conflicting literature exists on the chronic effects of drinking according to doctrine (e.g., 8*8) or ad libitum on physiological adaptations including fluid regulation, cardiovascular and metabolic fitness and hence on either health or performance outcomes.

Several problems exist with the hydration literature that could account for, and legitimise, the prevalent lack of adherence to drinking based on one’s change in body mass. Therefore, future research and doctrine-based guidelines must more extensively incorporate, verify and acknowledge the importance of the following: ecologically valid airflow (for exercise outdoors); many aspects of ordinarily available behaviours (e.g. thirst and self-regulation of exertional heat stress); blinding or full consideration for placebo effects of having water withheld before and/or during the exertional period of interest; the roles of free water deficit [16] and plasma osmolality [8] in these outcomes, particularly with different methods and timing of dehydration (before versus during the experiment); individual differences (e.g. aerobic fitness, genetics and beliefs about hydration); lack of familiarisation to the psychological stress of any imposed water deprivation [85]; the actual likelihood of serious heat illness in free-functioning individuals, particularly in non-competitive and non-military settings (i.e. the more typical situation for most people in the world); and the benefits and disadvantages for adaptation through both self-regulated but mild dehydration and drinking beyond thirst *during* routine aerobic training and heat acclimation.

## Abbreviations

ADH: anti-diuretic hormone; CNS: central nervous system; ECF: extra-cellular fluid; NSAID: non-steroidal anti-inflammatory drug; SIADH: syndrome of inappropriate anti-diuretic hormone secretion; TBW: total body water; USA: United States of America

## Competing interests

JDC and JKL declare that they have no competing interests. SNT is a consultant for Danone Waters France. PL is a minority shareholder of Teknicool Ltd., a New Zealand-based business that sells an ice slushie water bottle.

## Authors' contributions

Following discussion between JDC, SNT, JKWL and PBL, JDC produced a basic draft with equal contribution from SNT on sections 4 and 5. JDC, SNT, JKWL and PBL each contributed substantively to revisions and producing the final manuscript. All authors read and approved the final manuscript.
